# Intraspecific Variability of Stinging Nettle (*Urtica dioica* L.)

**DOI:** 10.3390/molecules28031505

**Published:** 2023-02-03

**Authors:** Sylwia Koczkodaj, Jarosław L. Przybył, Olga Kosakowska, Zenon Węglarz, Katarzyna B. Bączek

**Affiliations:** Department of Vegetable and Medicinal Plants, Institute of Horticultural Sciences, Warsaw University of Life Sciences WULS-SGGW, 159 Nowoursynowska Street, 02-776 Warsaw, Poland

**Keywords:** stinging nettle, intraspecific variability, populations, leaves, phenolic acids, flavonoids, chlorophylls, HPLC

## Abstract

The purpose of the work was to determine the intraspecific variability of the stinging nettle, in respect of the mass of leaves and their chemical composition, including the content of phenolic compounds and assimilative pigments. The objects of the study were 10 populations of nettle, originating from the eastern and southern part of Poland. The results obtained indicate a high level of variability between and within the populations investigated but not strictly related to their geographical locations. The mass of the leaves ranged from 0.19 to 0.28 kg dry weight (DW)/plant (Coefficient of variation (CV) = 16.33%). Using HPLC–DAD, four phenolic acids were detected, i.e., caffeoylmalic (570.97–1367.40 mg/100 g DW), chlorogenic (352.79–1070.83 mg/100 g DW), neochlorogenic (114.56–284.77 mg/100 g DW) and cichoric (58.31–189.52 mg/100 g DW) acids, with the last one differentiating populations to the highest degree (CV = 48.83%). All of the analyzed populations met the requirements of the European Pharmacopoeia (Ph Eur 10th) concerning the minimum content of caffeoylmalic and chlorogenic acids in nettle leaves (not less than 0.3%). Within the flavonoid fraction, two compounds were identified, namely rutoside (917.05–1937.43 mg/100 g DW, CV = 21.32%) and hyperoside (42.01–289.45 mg/100 g DW; CV = 55.26%). The level of chlorophyll a ranged from 3.82 to 4.49 mg/g DW, chlorophyll b from 1.59 to 2.19 mg/g DW, while the content of carotenoids varied from 2.34 to 2.60 mg/100 g DW. Given all the traits investigated, the level of a population’s polymorphism (CV) was visibly higher within a population than between populations. Population no. 4 was distinguished by the highest mass of leaves, and the highest content of rutoside, while population no. 2 was distinguished by the highest content of hyperoside, caffeoylmalic and chlorogenic acid.

## 1. Introduction

Stinging nettle (*Urtica dioica* L., Urticaceae family) is a wild-growing herbaceous perennial occurring almost all over the world. It grows mainly on damp and nitrogen-rich soil at partly shaded sites, including meadows, forests edges and brushwoods [1,2,3,4]. The nettle is highly polymorphic in terms of its ploidy level, developmental traits and chemical composition. Variation within the species is also manifested in its sexual system. Polygamy has been reported here, with several types of gynodioecy and various architectural types of monoecy [5]. So far, plenty of subtaxa, including subspecies, morpho- and ecotypes, varieties and hybrids have been recognized within the species [5,6]. Thus, it is treated as a complex taxonomic group described collectively as *U. dioica sensu lato* [1,4,7].

The species has been widely used all over the world. Both above- and underground organs are the source of many value-added products, used mainly in the pharmaceutical and the food industry. Leaves, roots and seeds are used for medical purposes; however, only the leaves (*Urticae folium*) and roots (*Urticae radix*) of the nettle are listed in the European Pharmacopeia (Ph Eur 10th) [4,8,9,10]. The leaves are rich in phenolics, especially phenolic acids. According to Ph Eur requirements, they should contain not less than 0.3% of the total of caffeoylmalic and chlorogenic acids [10]. The raw material contains other cinnamic acid derivatives, such as cinnamic, caffeic, *p*-coumaric, ferulic and sinapic acids, followed by hydroxybenzoic acid derivatives, namely gallic, gentisic, syringic, protocatechuic and vanillic acids [4,10,11,12,13]. Among flavonoids, apigenin, astragalin, genistein, isorhamnetin, kaempferol, rutoside, quercetin and catechin have been identified, while among coumarins, esculetin and scopoletin were detected [3,10,11,12,13]. Nettle leavesalsocontain a high amount of assimilative pigments, i.e., chlorophyll a and b, β-carotene, α- and β-xanthophyll (up to 1%) as well as vitamins C, B, K and mineral salts (mainly the salts of magnesium, potassium, calcium, iron and soluble silica). Traces of the essential oil and fatty acids have been detected in the aerial parts of the plant [4,8,10,11,12,13,14,15,16,17,18]. Given this wide spectrum of compounds, the nettle leaf indicates various biological activities, such as antioxidant, antibacterial, anti-inflammatory, anti-ulcer, anti-anemic, anti-asthmatic, and cardiovascular. The diuretic, hypoglycemic, immunostimulating, choleretic and metabolism-accelerating properties of the nettle leaf have been proved as well [3,12,17,18,19,20,21,22]. Due to their high nutritional value, fresh leaves, young herb and seeds have been used traditionally as a healthy food and feed. The textile industry uses sclerenchymatic fibers obtained from nettle stems to produce natural fiber, and leaf extracts for dyes. Moreover, nettle leaves or the whole herb are widely used in organic farming, e.g., as a natural fertilizer [2,4,10,23]. As mentioned above, underground organs (*Urticae radix*) of the species are also collected. Nettle root contains a complex of lipids and sterols (mainly β-sitosterol and stigmasterol), lignans, lecithin and polysaccharides. The raw material is mainly used in the phytopharmaceutical industry for the production of drugs applied in benign prostatic hyperplasia [4].

Due to the versatile possibilities of nettle application, it has recently become the object of interest of various industry sectors, especially the phytopharmaceutical, food or even paper industry. Taking into account global trends concerning healthy lifestyles, including functional food and supplement usage, as well as a tendency to “old herbs” return, nettle raw materials appear to be one of the most interesting herbal products, both for producers and consumers.

Up to now, nettle leaves have been mainly collected from natural sites. However, the extreme variability of the species, followed by its capacity to accumulate heavy metals and pesticides, may contribute to the fact that the raw material from wild-growing plants is often of questionable quality [23,24]. Given the increasing demand for nettle leaf, as well as recent requirements of the herbal industry, the need for large-scale cultivation of the plant has arisen. In the view of such need, one of the first steps to be taken is to recognize the range of natural diversity of the species, which is the main source of genes indispensable for future cultivation and agrotechnical programs. So far, only a few trials with nettle that address this problem have been undertaken [10,18,22,25,26]. Some of the experiments indicate that the most important traits in determining the variation amongst the species seem to be the height of plants, the length of internodes and the number of leaves per shoot [27]. Others show the diversity in the content of proteins, mineral salts and vitamins in the leaves of nettle ecotypes [23]. There is a lack of comprehensive studies that take into account the diversity among nettle populations both in terms of the mass of the obtained raw materials and their quality related to the requirements of the phytopharmaceutical industry. In our work, 10 nettle populations (each represented by 30 individuals) were assessed, specifically the mass of leaves and the content of phenolic acids in these raw materials, according to Ph Eur requirements. In order to exclude the influence of various environmental factors on the investigated plant traits, the research was carried out ex situ.

The aim of the work was to determine the intraspecific variability of wild-growing stinging nettles, in respect of the accumulation of biomass as well as phenolic compounds and assimilative pigments.

## 2. Results and Discussion

**Figure 1:**
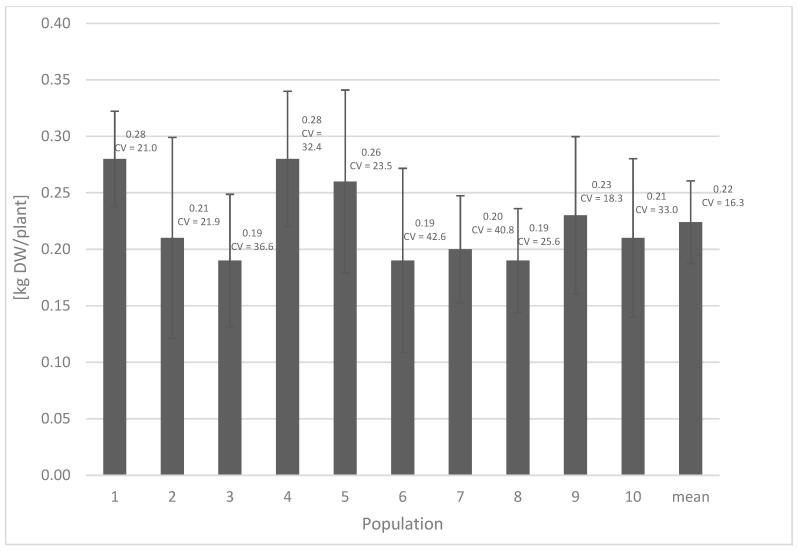
The mass of leaves, *n* = 30; CV: coefficient of variation; DW: dry weight.

The versatile use of the stinging nettle creates the need to obtain significant amounts of industrial raw materials. Currently, the collection of wild-growing plants does not meet the needs of the market, both due to the decreasing number of herb collectors and the variable quality of the raw materials obtained. Hence, there is a need to introduce the species into cultivation. The first step in this direction is to determine the range of variability and, furthermore, the potential of the selected forms for commercial herbal production. With regards to the nettle, this aspect has not been studied comprehensively. The available literature on the nettle concentrates on the methods of establishing plantations using different planting densities, mineral fertilization or the influence of plantation age on the raw material quality [23,25,28,29,30,31,32,33,34,35,36]. Our purpose was to determine the diversity of the species both within and between populations, to select individuals useful in further work on nettle propagation and industrial cultivation. The results obtained indicate the high polymorphism of the stinging nettle with regards to the mass of leaves and the content of phenolics and plant pigments, both between and within populations. The diversity regarding the mass of leaves between populations was distinctly lower (coefficient of variation (CV) = 16.3%) than the diversity among individual populations (CV from 18.3 to 42.6%). The mass of leaves ranged from 0.19 (pop. 3) to 0.28 (pop. 1 and 4) kg dry weight (DW) per plant. The results were presented in Figure 1. Jankauskiené [28] claims that this parameter may be even higher; it oscillates from 0.25 to 0.91 kg DW per plant. According to the literature data, the yield of nettle may be influenced by various factors, such as genetic, environmental (type of soil, temperature, humidity, water content, irradiance, photoperiod) and agricultural factors (e.g., nutrients and their availability; cultivation technology) [23,25,32]. Our work, carried out in uniform ex situ conditions, indicates that genetic factors are crucial here. This was also observed by Dumacheva et al. [37].

**Figure 2:**
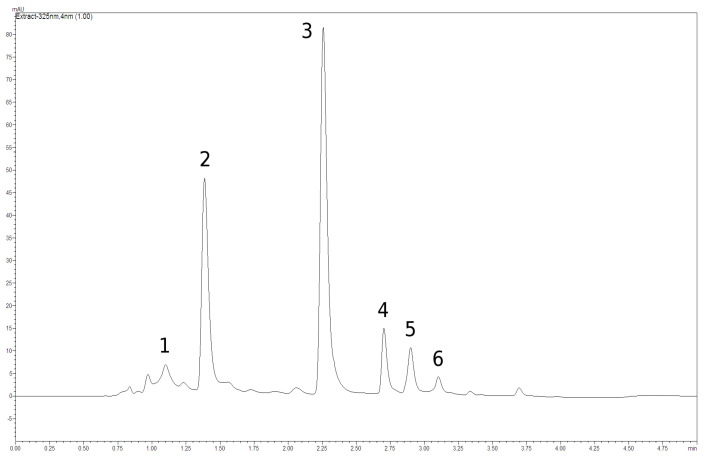
HPLC chromatogram of the nettle leaf methanolic extract: (**1**) neochlorogenic acid, (**2**) chlorogenic acid, (**3**) caffeoylmalic acid, (**4**) rutoside, (**5**) hyperoside and (**6**) cichoric acid.

**Figure 3:**
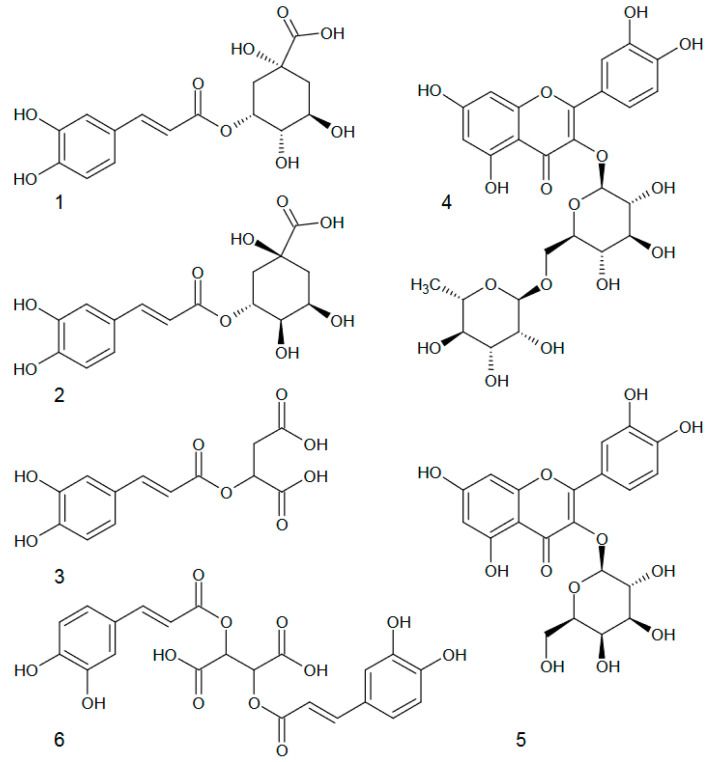
Chemical structures of identified phenolic compounds: (**1**) neochlorogenic acid, (**2**) chlorogenic acid, (**3**) caffeoylmalic acid, (**4**) rutoside, (**5**) hyperoside and (**6**) cichoric acid.

The content and composition of phenolics seem to be the most important quality parameter in nettle leaves. In our work, four phenolic acids were detected in the leaves, namely chlorogenic, neochlorogenic, caffeoylmalic and cichoric acids (Figure 2 and Figure 3, Table 1). These compounds represent depsides (cinnamic acid derivatives). Chlorogenic, neochlorogenic and cichoric acids constitute esters of caffeic and quinic acids, thus belonging to caffeoylquinic acid isomers. In turn, caffeoylmalic acid is an ester of caffeic and malic acids [38]. In our work, both caffeoylmalic and chlorogenic acids appeared to be the dominant ones (mean 945.7 and 595.1 mg/100 g, respectively), while neochlorogenic and cichoric acids were present in distinctly lower amounts (Table 1). All the investigated populations met the Ph Eur requirements concerning the minimum content of the sum of caffeoylmalic and chlorogenic acids (not less than 0.3%) [8]. However, they visibly varied in respect of the phenolic acid content, with cichoric acid as the most differentiated compound (CV = 48.8%). The amount of caffeoylmalic acid ranged from 571.0 to 1367.4 mg/100 g DW (CV = 23.9%), chlorogenic acid from 352.8 to 1070.8 mg/100 g DW (CV = 32.7%), neochlorogenic acid from 114.6 to 284.8 mg/100 g DW (CV = 24.0%), and cichoric acid from 58.3 to 189.5 mg/100 g DW (CV = 48.8%).

As regards diversity within a population, population no. 3 was the most variable in terms of caffeoylmalic acid content (CV = 43.1%), while population no. 4 had the biggest variation in chlorogenic acid (CV = 69.0%).

The results obtained correspond with the literature data. According to Grevsen et al. [29], the content of caffeoylmalic acid in nettle leaf ranged from 600 to 1710 mg/100 g DW, while chlorogenic acid from 250 to 1630 mg/100 g DW. In turn, Pinelli et al. [39] indicated lower amounts of these substances, i.e., 138.5 mg/100 g DW and 58.9 mg/100 g DW, respectively. So far, in nettle leaves, other phenolic acids have been detected, includingcaffeic, *p*–cumaric, cinnamic, ferulic, sinapic, syringic, quinic and protocatechuic acids [40,41,42,43]. It is worth noting that phenolic acids are considered to determine health-promoting values, since they reveal antioxidant, anti-inflammatory, antilipidemic, antidiabetic and antihypertensive activity [44,45].

In our work, two flavonoids were detected, namely rutoside and hyperoside. Both compounds belong to quercetin derivatives (Figure 2 and Figure 3, Table 2). The content of rutoside ranged from 917.1 (pop. no. 3) to 1937.4 mg/100 g DW (pop. no. 4) (CV = 21.3%), while the content of hyperosiderangedfrom 42.0 (pop. no. 1) to 289.5 mg/100 g DW (pop. no. 2) (CV = 55.6%). As regards the variability of both compounds within a population, high CV values were noticed in the cases of populations no. 1 and 5 (Table 2). The domination of rutoside in nettle leaves was observed earlier by Devkota et al. [17], Pinelli et al. [39] and Jeszka-Skowron et al. [40]. According to Zekowić et al. [41], among flavonoids, apigenin, luteolin, and naringenin and their derivatives are also present in this raw material.

The high chemical variability of the stinging nettle was previously reported by Repajić et al. [42] and Otles et al. [43]. This may be related to the physiological role of phenolics in plants. In general, the accumulation of such compounds is regarded as their adaptive response to environmental conditions including biotic and abiotic stresses. Light, temperature, salinity and heavy metal stresses stimulate the synthesis of both phenolic acids and flavonoids. Phenolics are also accumulated due to their inhibitory or toxic effects on nematodes, insects and herbivores [44,45]. Many authors also indicate the influence of the plant’s age and their phenological stage on the content of phenolic compounds [46,47]. In our investigation, the plants were grown ex situ, in order to eliminate the impact of selected environmental factors. Thus, it may be suspected that the observed diversity was determined mainly by genetic factors, including gender, since the species represent a subdioecious plants [6]. However, it should be proved by molecular (e.g., Next-Generation Confirmation—NGC) analysis in future research.

In the present work, the investigated populations were assessed with regard to the content of chlorophyll a and b, followed by the total carotenoids (Table 3). The content of chlorophyll a ranged from 3.8 to 4.5 mg/g DW (CV = 4.2%), chlorophyll b from 1.6 to 2.2 mg/g DW (CV = 8.9%) and the total carotenoids from 2.3 to 2.6 g/100 g DW (CV = 3.8%). The results obtained indicate a rather low diversity both between and within populations as regards the content of pigments mentioned above. According to Droštinová et al. [48], the contents of these compounds in the nettle are as follows: chlorophyll a, 6.7–8.2 mg/g DW; chlorophyll b, 2.3–3.0 mg/g DW; and carotenoids, 1.5–1.8 mg/100 g DW. Similar results were obtained by Durović et al. [13] and by Biesiada and Wołoszczak [30]. It is worth noting that in the case of stinging nettle, the content of chlorophyll a and b (besides its obvious role in photosynthesis) is important from the industrial viewpoint, since its leaves are used in chlorophyllin production. This substance is applied in the food and cosmetic industry as a green pigment [49,50].

## 3. Materials and Methods

### 3.1. Plant Material

The investigations were carried out at the experimental field of the Department of Vegetable and Medicinal Plants, Warsaw University of Life Sciences WULS-SGGW (52°10′18″ N; 21°05′23″ E), on alluvial soil (Table 4). The objects of the study were 10 wild-growing populations originating from southern and eastern parts of Poland (Table 5). The seeds from these populations were collected from natural sites in September 2020. In February 2021, they were sown into a peat substrate, in a greenhouse. In April, the seedlings were transplanted into multi-pots. At the beginning of June, well-rooted seedlings (60 per population) were planted out into the field at a spacing of 40 × 100 cm. The observations and the harvest of raw materials were performed the following year (2022). The herb was cut from 30 plants per population, at the vegetative stage of growth (the last ten days of June). The raw material was dried at 40 °C using a Memmert UFE 800 drying machine (Büchenbach, Germany). The leaves were separated from the stems, weighed, then powdered using a Herbal Medicine Disintegrator FW177 (Huanghua Faithful Instrument Co., Cangzhou, China), and subjected to chemical analysis (Section 3.2).

The climatic parameters of the 2022 vegetative season were recorded (Table 6).

### 3.2. Chemical Analysis

#### 3.2.1. Analysis of Phenolics by HPLC

The air-dried, powdered and homogenized samples (0.2000 g) were extracted with 250 mL of methanol (Sigma-Aldrich, Poznan, Poland) for 30 min at 40 °C in a sonication bath (Sonic 6, Polsonic, Warsaw, Poland). The extracts obtained were filtered into amber glass vials with a PTFE 0.22 μm pore and 25 mm diameter syringe tip filter (Sigma-Aldrich, Poznan, Poland).

The analytical work was performed using the Shimadzu Prominence chromatograph equipped with the SIL–20AC HT autosampler, SPD–M20A photodiode array detector and LC solution 1.21 SP1 chromatography software (Shimadzu, Kyoto, Japan).

The standards purchased from ChromaDex^®^ (Irvine, CA, USA) were separately dissolved with methanol in 25 mL volumetric flasks according to ChromaDex’s Tech Tip 0003: Reference Standard Recovery and Dilution, and used as standard stock solutions.

Diluting 10 µL and 100 µL of standard stock solutions with methanol in 10 mL volumetric flasks, 500 µL and 1000 µL in 5 mL volumetric flasks, as well as 1000 µL in 2 mL volumetric flasks, led to a series of working solutions. By injecting 1μL of these working solutions and the undiluted stock solution into the column in six replicates (*n* = 6), a six–point calibration curve was prepared. The parameters of the calibration curve were estimated in a spreadsheet (Office 365, Microsoft, Redmond, WA, USA) by analyzing the data obtained from the chromatography software. The signal-to-noise ratio approach were used to determine the limit of detection (LOD) (S/N of 3:1) and the limit of quantification (LOQ) (S/N of 10:1) (Table 7). Separations were achieved on a C18 reversed-phase column packed with 2.6 μm solid-core particles with a porous outer layer, measuring 100 mm × 4.60 mm (Kinetex™, Phenomenex, Torrance, CA, USA). A binary gradient of deionized water adjusted to pH 2 with phosphoric acid (mobile phase A) and ACN (mobile phase B) at a flow rate of 1.3 mL × min^−1^ was used as follows: 0.00 min—17% B; 0.50 min—17% B; 3.00 min—40% B; 3.10 min—40% B; 3.11 min—17% B; and 5.00 min. The oven temperature was set to 45 °C and the injection volume to 1 μL. The contents of the determined compounds were calculated as mg per 100 g of dry weight (DW) [51].

#### 3.2.2. The Content of Chlorophylls and Carotenoids

A total of 0.25 g of air-dried, powdered raw material was extracted by shaking with 30 mL of 80% aqueous acetone for 45 min at a room temperature. The solution obtained was filtered and filled up to 50 mL with the same solvent. The content of chlorophyll a, b and carotenoids was determined according to the method of Lichtenhaler and Welburn [52]. The absorbance was measured using a Schimadzu UV-1280 spectrophotometer (Schimadzu, Kyoto, Japan) at 470, 646 and 663 nm against a control sample of 80% acetone. Calculations of the amount of the pigments were completed by applying the following formula:

(1) $${Chl}_{a}=\frac{(12.21\times A_{663}-2.81\times A_{646})\times V}{1000\times m}$$

(2) $${Chl}_{b}=\frac{(20.13\times A_{441}-5.03\times A_{663})\times V}{1000\times m}$$

(3) $$Car=\frac{(1000\times A_{441}-3.27\times {Chl}_{a}-104\times {Chl}_{b})\times V\times 100}{229\times m}$$

A_x_—absorbance at x wavelength, m—mass of raw material, V—volume of used solvent Chl_a_—chlorophyll a, Chl_b_—chlorophyll b, Car—carotenoids.

The results of chlorophyll a and chlorophyll b content were expressed in mg/g, and carotenoids in mg/100 g.

### 3.3. Statistics

The results were statistically analyzed using one-factor analysis of variance (ANOVA) in Statistica 13.4.0 (2017). A post hoc Tukey’s (HSD) test with a significance level of α = 0.05 was used to compare groups. Homogeneous groups are marked by the same letters.

## 4. Conclusions

The results obtained in the present study showed that the intraspecific variability of stinging nettle is very high, both between populations and within them. Such a high level of polymorphism corresponds with the richness of the natural resources of the species. However, further investigations focused on the molecular basics of this diversity are needed. The populations examined in our work, especially those distinguished by a high yield of leaves and the content of biologically active compounds (populations no. 2 and 4.) may be used in future breeding and agrotechnical research. It is worth noting that in order to omit the variable character of stinging nettle, the plants selected for cultivation should be reproduced using the vegetative methods (by the cuts) instead of the generative ones (by the seeds). This may provide the homogenous and standardized raw material of known quality that is required by the herbal industry.

## Figures and Tables

**Table 1:** The content of detected phenolic acids (mg/100g DW).

Population	Neochlorogenic Acid		Chlorogenic Acid		Caffeoylmalic Acid		Cichoric Acid		Sum of Chlorogenic and Caffeoylmalic Acids	Sum of All Detected Phenolic Acids
		CV (%)		CV (%)		CV (%)		CV (%)		
1	204.5 ± 102.6	50.2	548.4 ± 234.0	43.8	1126.9 ± 288.3	25.6	189.5 ± 72.5	38.3	1675.3 ± 470.8	2069.3 ± 547.6
2	214.1 ± 65.6	30.6	1070.8 ± 503.6	47.0	1367.4 ± 431.8	31.6	184.3 ± 64.9	35.2	2438.2 ± 853.7	2836.6 ± 46.2
3	200.4 ± 78.2	39.0	513.6 ± 213.7	41.6	1114.7 ± 480.4	43.1	107.4 ± 47.9	44.6	1628.5 ± 803.2	1936.3 ± 79.9
4	150.5 ± 92.8	61.7	535.7 ± 367.5	68.6	801.7 ± 313.1	39.1	61.9 ± 21.7	35.1	1337.4 ± 750.1	1549.8 ± 16.2
5	242.7 ± 125.2	51.6	625.2 ± 315.4	50.4	851.7 ± 222.9	26.2	72.5 ± 31.2	42.9	1476.9 ± 533.4	1792.1 ± 82.6
6	284.8 ± 114.8	40.3	704.9 ± 300.8	42.7	1008.7 ± 288.2	28.6	85.0 ± 25.3	29.8	1713.7 ± 518.9	2083.4 ± 58.5
7	226.8 ± 84.0	37.0	578.5 ± 247.1	42.7	916.7 ± 225.4	24.6	86.8 ± 32.3	37.2	1495.2 ± 349.8	1808.8 ± 55.2
8	114.6 ± 62.6	54.6	601.6 ± 293.1	48.7	962.9 ± 303.2	31.5	72.2 ± 18.1	25.1	1564.5 ± 541.2	1751.2 ± 01.3
9	194.0 ± 61.4	31.7	419.1 ± 228.9	54.6	735.3 ± 242.6	33.0	72.4 ± 21.1	29.8	1154.3 ± 489.2	1420.7 ± 72.5
10	169.1 ± 54.7	32.3	352.8 ± 175.1	49.6	571.0 ± 235.5	41.2	58.3 ± 10.4	17.9	923.8 ± 413.7	1151.1 ± 413.8
Mean	200.1		595.1		945.7		99.0		1540.8	1839.9
CV	24.0		32.7		23.9		48.8		26.0	24.7

**Table 2:** The content of detected flavonoids (mg/100 g DW).

Population	Rutoside		Hyperoside		Sum
		CV * (%)		CV (%)	
1	1329.7 ± 845.10	63.6	42.0 ± 33.4	79.5	1246.4 ± 925.6
2	1375.9 ± 686.1	49.9	289.5 ± 170.8	59.0	1665.3 ± 816.7
3	917.1 ± 462.5	50.4	62.0 ± 50.7	81.9	873.3 ± 518.3
4	1937.4 ± 594.1	30.7	270.0 ± 167.4	62.0	2191.0 ± 698.1
5	1391.2 ± 851.1	61.2	114.8 ± 110.7	96.4	1270.0 ± 992.8
6	1479.9 ± 801.5	54.2	249.0 ± 128.9	51.7	1704.3 ± 895.8
7	1083.3 ± 512.1	47.3	99.8 ± 78.1	78.3	922.0 ± 711.6
8	1749.8 ± 661.2	37.8	160.2 ± 81.0	50.6	1910.0 ± 674.1
9	1223.3 ± 662.2	54.1	160.1 ± 99.0	61.8	1504.0 ± 682.9
10	1381.7 ± 601.0	43.5	115.3 ± 75.7	65.7	1368.9 ± 722.7
Mean	1386.9		156.3		1465.5
CV	21.3		55.6		28.5

* n = 30.

**Table 3:** The content of chlorophylls (mg/g) and carotenoids (mg/100 g DW).

Population	Chlorophyll a		Chlorophyll b		Total Carotenoids	
		CV * (%)		CV (%)		CV (%)
1	4.2 ± 0.6	14.7	1.9 ± 0.5	24.7	2.6 ± 0.4	14.6
2	3.8 ± 0.5	13.5	1.6 ± 0.4	23.3	2.3 ± 0.3	11.1
3	4.3 ± 0.3	8.0	1.9 ± 0.3	15.5	2.6 ± 0.2	7.6
4	4.1 ± 0.3	7.8	1.9 ± 0.4	19.9	2.5 ± 0.2	6.8
5	4.1 ± 1.1	26.0	2.0 ± 0.5	23.4	2.6 ± 0.3	11.4
6	4.2 ± 0.6	15.0	1.8 ± 0.5	25.5	2.5 ± 0.3	12.2
7	4.1 ± 0.6	14.8	1.9 ± 0.5	25.7	2.4 ± 0.3	13.0
8	4.1 ± 0.5	11.4	1.8 ± 0.3	18.6	2.4 ± 0.2	9.5
9	4.5 ± 0.6	12.6	2.2 ± 0.5	22.8	2.6 ± 0.2	8.8
10	4.1 ± 0.6	13.7	1.7 ± 0.4	22.2	2.4 ± 0.3	11.9
Mean	4.2		1.9		2.5	
CV	4.2		8.9		3.8	

* *n* = 30.

**Table 4:** Soil parameters * (pH and the content of main nutrients (mg/L)).

pH	NO_3_^−^	NH_4_^+^	P	K	Ca	Mg	Cl	Na	Cu	Fe	Mn	Zn
6.78	63	12	82	158	727	127	42	42	2.9	49.7	5.7	5.3

* samples for soil analysis were taken in February 2022.

**Table 5:** The geographical coordinates of origin of the studied nettle populations.

Population No.	Location	Voivodeship	Geographical Coordinates		Altitude
			Latitude	Longitude	
1	Bożejewo	Podlaskie	53°11′09.8″ N	22°17′45.3″ E	104
2	Grądy-Woniecko	Podlaskie	53°09′35.9″ N	22°23′29.8″ E	113
3	Świniary	Masovian	52°30′26.5″ N	22°15′65.2″ E	178
4	Sosnówek	Masovian	53°16′57.3″ N	20°58′47.6″ E	123
5	Łazy	Lubelskie	51°91′71.1″ N	22°42′11.5″ E	159
6	WolaSękowa	Subcarpathian	49°30′26.5″ N	22°00′30.9″ E	408
7	Karlików	Subcarpathian	49°26′06.3″ N	22°04′33.8″ E	478
8	Szczawne	Subcarpathian	49°24′14.4″ N	22°09′02.3″ E	394
9	Siedliska	Subcarpathian	49°57′22.3″ N	21°56′46.5″ E	229
10	Mszana	Subcarpathian	49°49′46.2″ N	21°64′84.7″ E	453

**Table 6:** The climatic parameters of 2022.

Months	Temperature (°C)	Rainfall (mm)	Air Humidity (%)	Sun Days
April	8	21.8	69	23
May	16	8.2	62	31
June	21	22.5	66	26
July	23	20.8	60	29
August	25	9.0	55	30
September	16	7.8	60	28
October	14	3.5	69	31

**Table 7:** HPLC–DAD validation parameters.

No.	Compound	Precision Intra–Day (CV%)	Precision Int er–Day (CV%)	Calibration Equation	R^2^ *	Linear Ran ge (mg/mL)	LOD ^a^ (µg/L)	LOQ ^b^ (µg/L)
1	5-*O*-caffeoylquinic acid (Neochlorogenic acid)	0.27	0.78	y = 1809.0 x − 1539.8	0.9999	0.39–392.0	18.39	61.31
2	3-*O*-Caffeoylquinic acid (Chlorogenic acid)	1.32	1.63	y = 6517.4 x − 12,016.6	0.9997	0.40–39.47	20.97	69.90
3	Caffeoylmalic acid	1.32	1.63	y = 6517.4 x − 12,016.6	0.9997	0.40–39.47	20.97	69.90
4	Quercetin 3-*O*-rutinoside (Rutoside)	0.37	0.86	y = 1434.0 x − 5093.0	0.9999	0.90–90.67	7.46	24.88
5	Quercetin 3-*O*-galactoside (Hyperoside)	1.25	2.14	y = 3435.5 x − 6882.2	0.9999	0.38–38.40	4.12	12.24
6	Cichoric acid	0.18	0.49	y = 3230.70 x + 6882.20	0.9998	0.46–456.96	11.47	38.23

* *n* = 6; ^a^ The limit of detection; ^b^ The limit of quantification.

## Data Availability

Not applicable.

## Sample Availability

Samples of the raw materials are available from the authors.
